# RhoA in aneurysmal subarachnoid hemorrhage

**DOI:** 10.18632/aging.101819

**Published:** 2019-02-09

**Authors:** Juan Ureña, María del Carmen González-Montelongo, Francisco Murillo-Cabezas

**Affiliations:** 1 Instituto de Biomedicina de Sevilla (IBiS), Hospital Universitario Virgen del Rocío/CSIC/Universidad de Sevilla, Dpto. de Fisiología Médica y Biofísica, Sevilla, Spain; 2 School of Biological Sciences, University of East Anglia, Norwich Research Park, NR4 7TJ, United Kingdom; 3 Instituto de Biomedicina de Sevilla (IBiS), Hospital Universitario Virgen del Rocío/CSIC/Universidad de Sevilla, Unidad de Neurocríticos, Sevilla, Spain

**Keywords:** subarachnoid haemorrhage, RhoA, biomarker, human mononuclear cells, cerebral vasospasm

Aneurysmal subarachnoid haemorrhage (aSAH) is a severe subtype of stroke, mediated by the spontaneous rupture of an intracranial aneurysm, that frequently occurs in people between 40 and 60 years of age. Cerebral vasospasm (CV), and related delayed cerebral ischemia (DCI), is a clinical syndrome of the focal neurologic deficits that develop typically three to fourteen days after the aneurysm rupture, and is a major cause of death and disability after aSAH. Vascular constriction and inflammation, more specifically leukocyte-endothelium interaction, appear to play a critical role in CV. Although the main focus of pharmacological treatment of aSAH is the prevention of DCI, the only pharmacological drug shown to reduce the risk of DCI and unfavourable outcome is nimodipine, an L-type Ca^2+^ channels (LTCCs) antagonist, which does not alter the incidence or severity of CV [1]. On the other hand, although inflammatory biomarkers that facilitate leukocyte-endothelium interaction have been found in the cerebral spinal fluid and serum of patients [2], none of these biomarkers have been shown to be useful tools for predicting CV development or outcome after aSAH. Therefore, it is necessary to study new pathophysiological pathways to improve outcomes and management of patients.

RhoA is a monomeric G-protein of the Ras superfamily that can be present in inactive (GDP-bound) and active (GTP-bound) conformational states to regulate cytoskeletal reorganization and cell polarity, a characteristic feature of migrating leukocytes. RhoA and its downstream effector, Rho-associated kinase (ROCK), can modulate the activity of myosin II, through inhibition of myosin light chain phosphatase, resulting in increased myosin regulatory light chain phosphorylation. In previous results from our laboratory, we described that depolarization-induced LTCCs activation triggers metabotropic Ca^2+^ release from the sarcoplamic reticulum, RhoA/ROCK activation and arterial sustained contraction [3]. In non-muscle cells, RhoA is thought to regulate the cytoskeletal rearrangement underlying leukocyte polarization and migration [4]. Since RhoA/ROCK has other cellular functions including the regulation of morphology, cell division, and gene expression, this signaling pathway is being investigated in other pathologies such as cancer, neurological disorders of the central nervous system and cardiovascular diseases [5].

Although it has been described that activation of ROCK in human peripheral blood mononuclear cells (PBMCs) is associated with cardiovascular pathologies, such as acute ischemic stroke, pulmonary arterial hypertension, and cardiovascular disease, its role in aSAH has not been studied. As RhoA/ROCK participates in sustained arterial contraction and leukocyte-endothelium interaction, we explored the role of RhoA in PBMCs from a small cohort of patients with aSAH [6]. We measured RhoA instead of ROCK to rule out effector activation by stimulus other than RhoA [7]. As a first step, we measured RhoA expression in PBMCs. We have shown that RhoA was significantly increased in PBMCs from aSAH patients on days 0, 2 and 4 versus healthy subjects and there was a significant correlation between RhoA expression and injury severity. As these patients are hospitalized immediately after bleeding, one possibility is that the increased RhoA expression could have been present before the haemorrhage occurred. There is evidence that suggests that leukocytes play a key role in the inflammatory response that leads to aneurysm formation and rupture. Future investigations should evaluate whether there is any correlation between RhoA in PBMCs and the presence of an aneurysm in patients. As the augmented amount of RhoA may facilitate protein activation in response to physical or chemical stimuli triggered by aSAH [2], we measured RhoA activity in PBMCs. The results showed that activated RhoA was increased on day 4 in PBMCs from patients that finally developed CV versus patients where vasospasm was absent. As it is known that CV begins three days after aneurysm rupture, activated RhoA could be evaluated, together with other biomarkers, to predict vasospasm in these patients.

While investigations continue to reduce angiographic vasospasm, research suggests that treatment of radiographic vasospasm is not always sufficient to improve clinical outcome. In addition to large vessel narrowing, aSAH leads to a number of microcirculatory changes. Thus more in vitro and in vivo studies of cerebral microcirculation are needed in order to understand the pathophysiology of aSAH, and to develop new therapeutics that target these microvessels and improve the clinical outcome [8]. Preliminary results described in González-Montelongo et al. [6] suggest that signaling pathways that regulate RhoA/ROCK may constitute therapeutic targets for treating both the sustained smooth muscle contraction and leukocyte-endothelium interaction. In fact, although studies analyzing the effect of hydroxyfasudil on CV prevention are limited, this ROCK inhibitor, approved for use in patients in Japan and China, reduces the occurrence of CV and cerebral infarction with significantly improved neurological prognosis in aSAH patients.

## References

[1] Macdonald RL, Schweizer TA. Lancet. 2017; 389:655–66. 10.1016/S0140-6736(16)30668-7 27637674

[2] Hong CM, et al. Biomarkers. 2014; 19:95–108. 10.3109/1354750X.2014.881418 24499240 PMC4346143

[3] Fernández-Tenorio M, et al. Circ Res. 2011; 108:1348–57. 10.1161/CIRCRESAHA.111.240127 21493898

[4] Sánchez-Madrid F, del Pozo MA EMBO J. 1999; 18:501–11. 10.1093/emboj/18.3.501 9927410 PMC1171143

[5] Loirand G, et al. Circ Res. 2006; 98:322–34 10.1161/01.RES.0000201960.04223.3c 16484628

[6] González-Montelongo MD, et al. Stroke. 2018; 49:1507–10. 10.1161/STROKEAHA.117.020311 29735721

[7] Silveira AA, et al. J Leukoc Biol. 2018; 103:87–98. 10.1189/jlb.3A0916-388RR 28798145

[8] Tso MK, Macdonald RL. Transl Stroke Res. 2014; 5:174–89. 10.1007/s12975-014-0323-4 24510780

